# Use of phytoestrogens and effects perceived by postmenopausal women: result of a questionnaire-based survey

**DOI:** 10.1186/1472-6882-14-262

**Published:** 2014-07-23

**Authors:** Anna Girardi, Carlo Piccinni, Emanuel Raschi, Ariola Koci, Benedetta Vitamia, Elisabetta Poluzzi, Fabrizio De Ponti

**Affiliations:** Department of Medical and Surgical Sciences, University of Bologna, via Irnerio 48, I-40126 Bologna, Italy

**Keywords:** Phytoestrogen, Food supplements, Pattern of use, Postmenopausal symptoms, Perceptions

## Abstract

**Background:**

Use of food supplements-containing phytoestrogens among postmenopausal women is rapidly increasing. Although phytoestrogens are often perceived as safe, evidence for overall positive risk-benefit profile is still inconclusive. The chance to buy them by user’s initiative does not facilitate surveys on their prevalence and pattern of use. The aim of this study was to describe the pattern of use and self-reported positive and negative perceptions of phytoestrogens in post-menopausa.

**Methods:**

A questionnaire was administered to women who were buying food supplements containing phytoestrogens in 22 pharmacies located in the Bologna area (400,000 inhabitants). Questionnaire was structured into 3 sections: (a) socio-demographic information, (b) pattern of use, (c) positive and negative perceptions.

**Results:**

Data on 190 peri- and post-menopausal women (aged 38–77) were collected. Women stated to use phytoestrogens to reduce hot flushes (79%), insomnia (15%), mood disturbances (14%) and prevent osteoporosis (15%). The majority (59%) took phytoestrogens routinely, whereas 28% in 3-month cycles. Among positive perceptions between short- and long-term users, a not negligible difference was reported for relief of hot-flushes (68% in short-term vs. 81% in long-term users; p = 0.04). Negative perceptions were reported more frequently in the long-term group, and this difference was statistically significant for edema (6% in short-term vs. 17% in long-term users; p = 0.04), but not for other effects: e.g., swelling sensation (10% vs. 21%; p = 0.09), somnolence (7% vs. 10% p = 0.62), fatigue (4% vs.11% p = 0.15).

**Conclusions:**

In the Bologna area, the pattern of use of phytoestrogens for menopausal symptoms is heterogeneous, and women overall find these substances to be beneficial, especially for relief of hot-flushes. Other positive perceptions decreased with long-term use. Negative perceptions, especially estrogen-like effects, seem to be infrequent and increase with long-term therapy. Physicians should pay attention to effects perceived by post-menopausal women and routinely monitor the use of phytoestrogens, in order to recognize possible adverse effects and actual benefits.

**Electronic supplementary material:**

The online version of this article (doi:10.1186/1472-6882-14-262) contains supplementary material, which is available to authorized users.

## Background

The peri- and post-menopausal years are typically characterized by vasomotor symptoms, such as hot flushes and night sweats, due to the reduction of circulating estrogen levels. Therefore, the Hormone Replacement Therapy (HRT) approach, by replacing endogenous estrogens with exogenous hormones, was primarily advocated to limit both short and long-term consequences due to decreased estrogen level. In fact, several Randomized Control Trials recruiting postmenopausal women demonstrated the reduction of vasomotor symptoms in HRT short-term users [1].

The HRT was considered the most common choice for the treatment of menopausal symptoms until the publication of Women Health Initiative (WHI) Trial, in 2002. This trial reported that the risk of HRT treatment in the long-term use outweighed the benefits, due to the increased risk of breast cancer, stroke, thromboembolism, gallbladder disease and dementia [2]. Moreover, results from the WHI follow-up study did not support use of HRT for chronic disease prevention, although it remains a reasonable option for the management of moderate to severe menopausal symptoms [3].

Although the HRT is still considered the most effective treatment for unacceptable vasomotor symptoms, their prescription rates drastically decreased after the publication of WHI results [4] and after the new recommendations published by Regulatory Agencies [5, 6]. The use of alternative therapies for menopausal symptoms became very popular among women; in particular, therapies based on phytoestrogens represent the most common alternative to HRT [7].

Phytoestrogens are non-steroidal compounds derived both from plants and from the *in vivo* metabolism of precursors contained in several plants traditionally used as food. These secondary metabolites induce biological responses and can mimic or modulate the actions of endogenous estrogens, usually by binding to Estrogen Receptors [8]. The most common types of phytoestrogens are coumestans, lignans and isoflavones.

The potential benefits of therapies based on phytoestrogens have been investigated during the last two decades with escalating interest, as demonstrated by the increasing number of pre-clinical and clinical trials since the 90s [1]. A number of Randomized Controlled Trials have been conducted in menopausal women to verify the efficacy of phytoestrogen in improving menopausal symptoms [7]; however, there is still a lack of good information on the risk and benefits of this approach.

The classification of the phytoestrogen products as food supplements does not facilitate surveys on their prevalence and pattern of use by collecting prescription data: it is reported that 70% of women taking dietary supplements do not inform their doctors [9]. Therefore, direct dispensation to the patient by the pharmacists represents the most suitable observational point to collect data on this topic.

The purpose of this study was to describe the pattern of use of phytoestrogens in peri- and post-menopausal women and to report their positive and negative perceptions.

## Methods

A questionnaire-based survey was conducted in 22 Pharmacies located in the Bologna area (400,000 of inhabitants) and enrolled all women who were buying food supplements containing phytoestrogens between April and September 2012. The questionnaire was distributed by the pharmacist, making sure to avoid influence on the free choice of the product and answers to relevant questions. After signing informed consent, women autonomously filled-in the questionnaire, with the pharmacists assistance in case clarifications were needed.

The questionnaire was anonymous and structured in three different sections: (1) socio-demographic information (e.g. date of birth, nationality, work position); (2) pattern of use (e.g. duration and frequency of therapy); (3) positive and negative perceptions. At the end of the questionnaire, the name of the product was recorded (Additional file 1: Questionnaire).

The study obtained the approval from the Ethic Committee of the Local Health Authority of Bologna.

All collected data were analysed by using descriptive statistics. Subjects were grouped according to treatment duration in short-term users (≤1 yr of treatment) and long-term users (>1 yr of treatment). Differences in demographic and clinical characteristics, as well in positive and negative perceptions between these two groups were assessed by appropriate statistical tests (as acknowledged in the relevant tables; p <0.05).

## Results

We collected 190 questionnaires from 38- to 77-year old women. The 88% of the cohort declared to be in the postmenopausal period (41% within the previous 4 years); 35% of participants reported high cholesterol levels, 18% hypertension, 11% other vascular disorders and 4% breast cancer diagnosis before starting phytoestrogens (Table 1).

**Figure 1:**
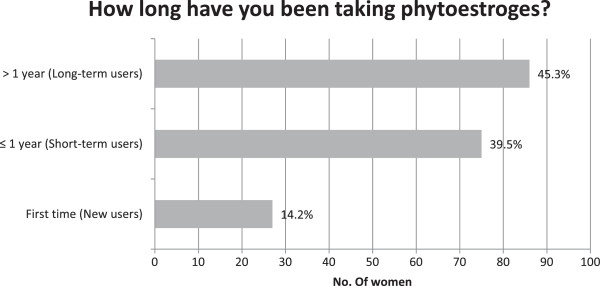
**Period of administration of phytoestrogens.** Missing data on duration of therapy in 2 questionnaires.

In 45.3% of cases, women have been taking phytoestrogens for more than 1 year (long-term users), 39.5% for less than 1 year (short-term users) and the 14.2% declared to be new user (i.e., they resorted to the product for the first time) (Figure 1). No differences in demographic and clinical characteristics were found between short- and long-term users, except for time-related variables (age and menopausal status).

**Table 1:** **Socio-demographic and clinical characteristics of responders**

Features	Total	Short-term users (≤1 year)	Long-term users (>1 year)	Difference between short- and long-term users	New users
	N (%) 190 (100)	N (%) 75 ^a^ (100)	N (%) 86 ^a^ (100)	p ^b^	N (%) 27 ^a^ (100)
***Age***					
Average	57	55	58	0.00***	56
Range	38-77	45-76	38-74		45-77
***Nationality***					
Italian	180 (97)	72 (96)	85 (99)	0.22	25 (93)
Not Italian	6 (3)	3 (4)	1 (1)	0.34	2 (7)
***Concomitant Disease***					
High blood pressure	45 (24)	16 (21)	21 (24)	0.83	8 (30)
Diabetes	4 (2)	1 (1)	1 (1)	insufficient observations	2 (7)
Osteoporosis	24 (13)	8 (11)	14 (16)	0.75	2 (7)
Heart disorders	18 (9)	5 (7)	9 (10)	0.58	4 (15)
Circulatory disorders (es. thrombosis)	27 (14)	11 (15)	13 (15)	1.00	3 (11)
High levels of Cholesterol	87 (46)	34 (44)	43 (50)	0.60	11 (41)
Liver diseases	7 (4)	4 (5)	3 (3)	0.71	0 (0)
Hepatic lithiasis (calculus)	17 (9)	6 (8)	8 (9)	0.95	3 (11)
Obesity	18 (9)	6 (8)	8 (9)	0.95	4 (15)
***Concomitant Diagnosis***					
Fracture of the thigh-bone or spine	3 (2)	2 (3)	1 (1)	0.60	0 (0)
Breast cancer	8 (4)	1 (1)	6 (7)	0.12	1 (4)
Uterine cancer	4 (2)	3 (4)	1 (1)	0.34	0 (0)
Ovarian cancer	1 (1)	0 (0)	1 (1)	insufficient observations	0 (0)
***Family history of breast cancer***	36 (80)	4 (5)	16 (19)	0.02**	6 (22)
***Already in the menopausal period***	166 (87)	60 (80)	84 (98)	0.00***	21 (78)

^a^Missing information on therapy duration in 2 questionnaires; ^b^Two-sample T-test was used to compare average age, Exact Fisher test was used in case of variables with 5 obs. or less, Two-Sample test of proportions was used for other variables; ***p-value < 0.01; **p-value < 0.05.

Among long-term users, 60 specified the duration of the therapy: 40 for less than 5 years, 17 for 6–10 years and 3 for more than 11 years. Fifty-two percent of women were advised to take phytoestrogens by specialist (gynecologist or endocrinologist), 19% by pharmacist, and 9% by general practitioner.

Twenty-five different products containing phytoestrogens were considered in the survey; all products contained isoflavones (dosage ranged 40 - 80 mg), mainly derived from soy (56%) and *Trifolium prantense* (28%).

**Figure 2:**
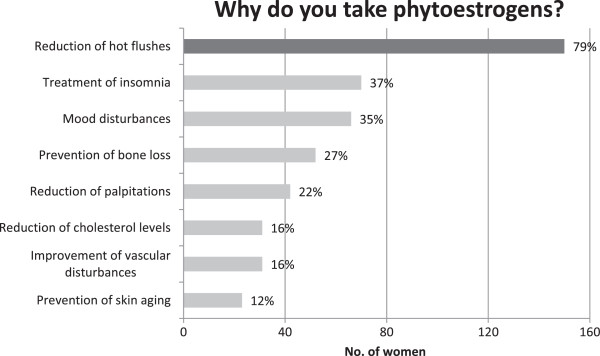
**Benefits expected by women using phytoestrogens.**

Participants used phytoestrogens with different goals, in particular to reduce hot flushes (79%), insomnia (15%), mood disturbances (14%), and to prevent osteoporosis (15%; Figure 2). Concerning the pattern of use, the majority (59%) took their food supplement routinely, every day, whereas 28% in cycles (especially 3-month-cycles).

Seventy-four percent of the participants perceived a reduction of hot flushes with the phytoestrogen assumption; positive perceptions included also increased well-being (52%), mood improvement (48%) and reduction of palpitations (37%). Among negative perceptions, 15% of women reported a swelling sensation, 11% edema occurrence, 8% somnolence and 7% fatigue.

The comparison in terms of positive perceptions between short-term and long-term users showed that: reduction of hot flushes for long-term users was greater (81% vs. 68%, p = 0.04), whereas no statistically significant difference was found in reduction of palpitation (37% vs. 37%, p = 0.92) and mood improvement (51% vs. 48%, p = 0.89; Table 2). Negative perceptions were reported more frequently among long-term users; differences were statistically significant for edema (6% in short-term vs. 17% in long-term users; p = 0.04), but not for other effects: swelling sensation (10% vs. 21%; p = 0.09), somnolence (7% vs. 10% p = 0.62), fatigue (4% vs.11% p = 0.15; Table 3).

**Table 2:** **Differences in positive perceptions between short- and long-term users**

Positive Perceptions	All users N (%)	Short-term users N (%)	Long-term users N (%)	Chi square	p value
	**163** ^**a**^ **(100)**	**75** ^**b**^ **(100)**	**86** ^**b**^ **(100)**		
Reduction of hot flushes	121 (74)	51 (68)	70 (81)	4.34	0.04
Well-being increase	84 (52)	44 (59)	40 (47)	0.41	0.52
Mood increase	79 (48)	38 (51)	41 (48)	0.02	0.89
Palpitation	60 (37)	28 (37)	32 (37)	0.01	0.92

^a^Excluding new users; ^b^Missing data on duration of therapy in 2 questionnaires.

**Table 3:** **Differences in negative perceptions between short-term and long-term users**

Negative Perceptions	All users N (%)	Short-term users N (%)	Long-term users N (%)	Chi square	p value
	**163** ^**a**^ **(100)**	**67** ^**b**^ **(100)**	**82** ^**b**^ **(100)**		
Swelling sensation	24 (15)	7 (10)	17 (21)	2.89	0.09
Edema	18 (11)	4 (6)	14 (17)	4.38	0.04
Somnolence	13 (8)	5 (7)	8 (10)	0.24	0.62
Fatigue	12 (7)	3 (4)	9 (11)	2.10	0.15

^a^Excluding new users; ^b^Missing data on duration of therapy or negative perceptions in 12 questionnaires.

## Discussion

This study provides for the first time Italian data about the pattern of use of phytoestrogens for postmenopausal symptoms and relevant perception of effects by users: the majority of women took this food supplement every day for more than 1 year, especially to reduce vasomotor symptoms. Overall, women found these substances to be beneficial, especially for relief of hot-flushes; this effect seemed to increase with long-term use, although these findings could partially be ascribed to physiological reduction of vasomotor symptoms during the menopausal course. On the contrary, other positive perceptions (either physical and psychological) appeared to decrease with long-term use. Negative perceptions (e.g., swelling and edema) seem to be infrequent, increase with long-term therapy and, notably, to be estrogen-related. It is well known that phytoestrogens structurally resemble estradiol and because of this similarity, they can bind human Estrogen Receptors and mimic the actions of endogenous estrogens [9]. Healthcare professionals should be actively involved in monitoring women exposed to phytoestrogens. In particular, both physicians and pharmacists should critically consider if and when therapy is appropriate. Moreover they have a crucial role in recognizing possible clinical consequences, either benefits and adverse effects, of phytoestrogens also by paying attention to women perceptions.

This investigation has different limitations, including population enrollment, as women were involved on a voluntarily basis and the study was limited to the Bologna area. In addition, the qualitative and quantitative composition of the products were heterogeneous and not exactly titrated, therefore reported perceptions could be also affected by different doses and ingredients of products. Our results were based on women perceptions, that cannot be considered a hard endpoint.

Despite limitations, this approach was capable of detecting some expected estrogen-like effects by using proxy measures (e.g., swelling and edema), and it is feasible and replicable as a possible strategy when formal studies are not easily applicable, as in the case of food supplements. Furthermore, this strategy is also able to describe women’s attitude, which is a direct consequence of the source of the suggestion to take phytoestrogens.

## Conclusions

Studies conducted so far did not fill the gap of knowledge to support recommendations about appropriate use of phytoestrogen. Future efforts should be addressed towards clinical trials with adequate sample size and designed to compare head-to-head different concentration of phytoestrogens against active comparator, namely hormone therapy or any other standard therapy for a given disorder.

Moreover, our survey suggests that it is possible to obtain data about extent and pattern of use of phytoestrogens, with the collaboration of pharmacists. Women’s perceptions should be considered as an important endpoint while investigating the use of food supplements, especially because the conduction of formal studies is challenging in this category of products. Our questionnaire could be a useful tool for this purpose and should be adopted in additional cohorts of women to verify our results on a more extended area.

## Electronic supplementary material

Additional file 1: **Questionnaire.** (PDF 468 KB)
