# Supplementary material for: Use of phytoestrogens and effects perceived by postmenopausal women: result of a questionnaire-based survey
Source: BMC Complement Altern Med. 2014 Jul 23;14:262. doi: 10.1186/1472-6882-14-262 (PMC4117950; doi:10.1186/1472-6882-14-262)
Supplement: Supplementary file 1 — Additional file 1: Questionnaire. (PDF 468 KB) [file 12906_2014_1831_MOESM1_ESM.pdf]

Supplementary Material

Use of phytoestrogens and effects perceived by postmenopausal women: results of a questionnaire-based survey

QUESTIONNAIRE*

PART 1 - GENERAL INFORMATION

1. **Date of Birth** |__|__|__|__|

Height|__|__|__| cm

Weight |__|__| kg

2. **Nationality**

☐ Italian

☐ Not Italian (specificify)________________________________

(How many years have you been in Italy?__________________)

3. **Marital Status**

☐ single

☐ married or cohabiting

☐ widow

☐ separated or divorced

4. **How many children do you have?**

☐ 0

☐ 1

☐ 2

☐ more than 2

5. **With whom do you live?**

☐ alone

☐ housband or partner

☐ children

☐ other relatives (parents,..)

☐ other persons (not relatives)

6. **What is your educational level?**

☐ none

☐ elementary school

☐ high school

☐ bechelor degree (in ____________________)

7. **What is your current work position?**

☐ self-employed

☐ employee

☐ unemployed

☐ houswife

☐ retired

PART 2 - PATTERN OF USE

8. **Who advised you about phytoestrogen-containig food supplement?**

☐ friends

☐ advertisement

☐ general practitioner

☐ pharmacist

☐ specialist (gynecologist or endocrinologist)

☐ other ____

9. **If the advisor was someone different from general practitioner, did you tell him about the use of this product?**

☐ Yes

☐ No If no, why? ____

10. **Why do you take phytoestrogens? (Multiple choice)**

☐ to prevent osteoporosis

☐ to reduce circulating cholesterol levels

☐ to improve venous circulation

☐ to reduce palpitations

☐ to reduce hot flushes

☐ to prevent skin aging

☐ to improve mood

☐ to treat insomnia

☐ other ____

11. **Are you already in the postmenopausal period?**

☐ Yes If yes, from how long? __________________________________

☐ No

12. **How long have you been taking the phytoestrogen?**

☐ this is the first time I bought it (new user) ***[please do not fill-in questions 14, 18, 19 and 20]***

☐ less than 1 month

☐ 1 – 6 months

☐ 6 months – 1 year

☐ more than 1 year (specify)____

13. **For how long do you think you will take phytoestrogen?**

☐ forever

☐ for a defined period (specify)____

☐ it depends on benefits and side effects

14. **How many times do you take the phytoestrogen?** ***[Please not fill-in if you are new user]***

☐ in cycles

If yes, please specify

1) cycle length
- ☐ 1-month-cycle
- ☐ 2-months-cycle
- ☐ 3-months-cycle
- ☐ more than 3 months

2) interval between cycles
- ☐ 2-3 weeks
- ☐ 1 month
- ☐ 2 months
- ☐ more than 2 months

☐ routinely

If yes, please specify
- ☐ once daily
- ☐ twice daily
- ☐ more than once a week
- ☐ less than once a week
- ☐ other _______________

Please answer Yes or No to each item

15. **Do you suffer from or have you ever suffered from**

	**Yes**	**No**
High blood pressure	☐	☐
Diabetes	☐	☐
Osteoporosis	☐	☐
Heart disorders	☐	☐
Circulatory disorders (es. thrombosis)	☐	☐
High levels of Cholesterol	☐	☐
Liver diseases	☐	☐
Hepatic lithiasis (calculus)	☐	☐
Obesity	☐	☐

Please answer Yes or No to each item

16. **Do you have history of:**

	**Yes**	**No**
Fracture of the thigh-bone or spine	☐	☐
Breast cancer	☐	☐
Uterine cancer	☐	☐
Ovarian cancer	☐	☐

Other cancer ____

17. **Did someone in your family suffer from breast cancer?**

☐ Yes If yes, how old was she/he at the time of the diagnosis and which was the outcome? _______________________

☐ No

Please answer Yes or No to each item

18. **After starting the treatment, have you perceived:** ***[Please not fill-in if you are new user]***

	Yes	No
Reduction of hot flushes	☐	☐
Reduction of palpitations	☐	☐
Improvement of venous circulation	☐	☐
Mood improvement	☐	☐
Weight loss	☐	☐
Well-being improvement	☐	☐

Other positive perception_________________________________

Please answer Yes or No to each item

19. **Your laboratory analysis, if available, showed:** ***[Please not fill-in if you are new user]***

	Yes	No
Increase of bone mineral density (BMD)	☐	☐
Reduction of total cholesterol levels	☐	☐
Reduction of glycemia (blood sugar)	☐	☐

Other ____

Please answer Yes or No to each item

20. **Which of the following negative perceptions have you noticed so far?** ***[Please not fill-in if you are new user]***

	Yes	No
Fatigue	☐	☐
Somnolence	☐	☐
Nausea and vomiting	☐	☐
Diarrhea	☐	☐
Swelling sensation	☐	☐
Oedema	☐	☐
Headache	☐	☐
Abnormal bleedings	☐	☐
Thyroid disorders	☐	☐

Other ____

21. **Comments on personal feelings** (other than what already declared)

____

____

____

NAME OF THE PRODUCT ____

(Please attach copy of the product's label)

** Translated from the original Italian version*
